# Impact of COVID-19 Pandemic on Women’s Rights and Wellbeing: Analysis of the Ugandan Response to the Global Virus

**DOI:** 10.1007/s41134-022-00229-w

**Published:** 2022-10-26

**Authors:** Hadijah Mwenyango

**Affiliations:** School of Health & Social Care, Edinburgh Napier University, Sighthill Campus, Edinburgh, EH11 4BN Scotland

**Keywords:** COVID-19, Lockdown, Gender, Women and girls, Rights, Wellbeing

## Abstract

The COVID-19 pandemic caused dilemmas for the most vulnerable populations around the world. This article describes the gendered effects of the pandemic on Ugandan women’s rights and wellbeing and provides suggestions for local and international practice. Mandatory lockdowns and movement restrictions created negative implications for women’s attainment of economic, social, cultural, political and civil rights and intensified pre-existing gender inequalities between women and men. The findings of intensified gender inequities, gender-based violence, sexual abuse, scanty access to reproductive health services and social justice, and barriers to participation in education, employment and politics indicate that response measures were not aligned with the government’s legal and policy framework for addressing gender inequities. This research indicates that governments, civil society organisations and the international community must undertake proper gender analysis in designing response measures and guidelines not only for COVID-19 but also in other emergencies. All response measures during emergencies must be coordinated, monitored and evaluated to ensure efficient and effective protection of the vulnerable and conformity to human rights standards.

## Introduction

The Sustainable Development Goals (SDGs)—a plan to achieve 17 goals with 169 targets in 15 years evolved in 2015. These cover all aspects to transform lives. The goals provide hope that the world can transcend biting poverty, food insecurity, unhealthy lives and discriminatory gender practices and achieve equality and women empowerment and sustainable and inclusive development. The corona virus disease (COVID-19) pandemic emerged while international partners were struggling to redress imbalances as set out in the SDG plan to secure good futures for their populations. This global enemy was declared a public health emergency by the World Health Organisation (WHO) on 30 January 2020.

In March 2020, Uganda reported its first confirmed COVID-19 case and cases gradually increased to 163,301 as of March 2022 (Ministry of Health, 2022). Given the collective risk, the government of Uganda (GOU) announced a lockdown and a country-wide curfew to control the spread of COVID-19. Although the response measures served their purpose (curbing the spread of COVID-19 in the general population), the impact of these rules has reversed the country’s little achievements in the protection and promotion of women’s rights and wellbeing.

Worldwide, the COVID-19 pandemic has painted a clear contribution of women to the care and wellbeing of households and exposed their vulnerability to gendered discrimination (United Nations (UN) Women, 2020). The lockdowns in various countries have intensified gender inequality in various sectors—health, economy, security and social protection (UN Women, 2020). Women and girls have suffered gender inequities including forced marriage, gender-based violence, sexist policies and barriers to participation in education and employment (Population Matters, 2021). Women’s enjoyment of human rights including economic, social and cultural rights has also been curtailed (Lutamaguzi & Nannozi, 2020). Extremely affected women include the poor without income or savings, hard-to-reach and stigmatised (such as those in rural areas), adolescents, sex workers, refugees and people with disabilities (The Program on Global Health Justice and Governance (PGHJG) and Bukuluki 2021; United Nations (UN), 2020).

Based on a comprehensive literature review, this article describes the impact of the pandemic on women’s rights and wellbeing. It explains the recurrence of gendered inequalities from the COVID-19 crisis. This research contributes to global attempts for strengthening women’s capacities and rights. It is expected that national response teams across sectors will consider the gendered implications of COVID-19 and put in place a broad and gender-responsive COVID-19 prevention and response plan to reduce conditions of vulnerability and strengthen resilience across various disadvantaged groups.

The article begins with a brief description of methods and provides the legal and policy context of the rights and management of COVID-19 in Uganda. The second part analyses the gendered effects of COVID-19 on women’s rights and wellbeing under six themes: the implications of COVID on women’s economic rights, COVID-19 and its effects on women’s unpaid work, COVID-19 and women’s health needs, COVID-19 and gender-based violence (GBV), COVID-19 implications on the young women’s right to education and COVID-19, discrimination and the legal protection of women’s rights. The final section provides local and international implications for practice.

## Situation Analysis of Women’s Rights and Gender Equality

Uganda has made some advancements in the achievement of women and girls’ rights in the political, economic and social spheres. For instance, the Universal Primary Education (UPE) programme increased the overall enrolment of women from 2.7 million in 1995 when it was rolled out to the recent 4. 4 million in 2017 (Uganda Bureau of Statistics (UBOS), 2019). Though the comparison of the Gender Parity Index (GPI) between 2012/2013 and 2016/2017 indicates existing inequality in favour of males being enrolled in primary and secondary education levels (UBOS, 2018), there has been a gradual improvement in the enrolment of girls and women in literacy programmes. The glimpse of hope is that the GOU is aiming at reducing vulnerability, gender equality and the gender gap index from 0.523 in 2017 to 0.5 in 2025 (National Planning Authority (NPA), 2020).

Women have progressively become visible in leadership and public decision-making. The Ugandan Constitution provides for the reservation of one seat for a woman member of parliament for every district and at least one-third of local council seats are reserved for women. Women's reproductive health packages have also been promoted by the government and women’s organisations and this facilitates their participation in the productive economy and ability to meet their survival needs. For example, the total fertility rate declined from seven to five children per woman between 1995 and 2016 (UBOS, 2019).

Despite these achievements, research indicates high school dropout rates for girls in primary and secondary education, increased gender disparities in enrolment and completion rates and more women than men in low-paid occupations (NPA, 2021; UBOS, 2019). Besides, although women are represented in parliament and other political offices, the proportion of women in leadership is still low compared with that of men. This is compounded by the lack of political will, patriarchal cultures (Gardsbane et al., 2021) and absence of monitoring and evaluation of the policies and programmes in redressing gender imbalances. The emergence of COVID-19 not only exacerbated pre-existing inequalities but also triggered further challenges in an already fragile situation (UN, 2020).

## Legal and Policy Context

Uganda has a rich legal framework that addresses gender-responsive planning and programme design at the national, regional and international levels. Uganda’s Constitution guarantees equality of all people under the law in all spheres of political, social and cultural life and the enjoyment of equal protection by the law in all aspects. Chapter four of the constitution lays down fundamental rights and freedoms of women and fair representation of marginalised groups, freedom from discrimination, and proposes affirmative action in favour of marginalised groups based on gender or any other reason created by tradition or customs (Government of Uganda, 2006).

The Directorate of Gender & Community Development under the Ministry of Gender Labour and Social Development (MoGLSD) is mandated to spearhead and coordinate sectors to ensure that the concerns and experiences of women and men are fully integrated into the design, implementation and monitoring and evaluation policies and programmes.

With this initiative, the GOU has mainstreamed gender issues in several policies and programmes including, the Gender Equity Budgeting Policy, the National Priority Gender Equality Indicators (2016), the National Policy on the Elimination of Gender-based Violence in Uganda, the Uganda Vision 2040, the Third National Development Plan (NDPIII) 2020/21 – 2024/25, the Uganda Gender Policy 2007 (UGP), the Gender in Education Policy, the Education Sector Strategic Plan (ESSP) 2007–2015 and the Business, Technical, Vocational Education and Training (BTVET) Strategic Plan (2012/3- 2021/2) to improve the status of women and girls. For instance, Gender Education Policy promotes the implementation of universal and compulsory education, engendered education curricula, gender equality in teacher recruitment, and deployment, sex education, re-entry into formal education for young mothers and advocacy for stay-in-school campaigns for girls (Ministry of Education and Sports (MoES), 2016).

In addition, Uganda is a signatory to regional instruments that promote gender equality and empowerment including the East African Community (EAC) Treaty (2000), the Common Market for Eastern and Southern Africa (COMESA) Gender Policy (May, 2002), the Protocol on the Rights of Women in Africa (July, 2003), the Inter-Government Authority on Development (IGAD), Gender Policy and Strategy (July, 2004), the New Partnerships for African Development (NEPAD) and the AU Heads of State Solemn Declaration on Gender Equality (July, 2004). Internationally, the country ratified the Convention on Elimination of All Forms of Discrimination Against Women (CEDAW, 1979), the Beijing Declaration and Platform for Action (1995), the International Conference on Population and Development (1994) and the United Nations Declaration on Violence Against Women (DEVAW, 1993).

This allegiance obliges the government to enact programmes to enhance women’s human rights and wellbeing. In 1997, the government enacted a National Gender Policy (NGP) which was revised in 2007. The NGP presents strategies focused on developing and implementing livelihood interventions that respond to diverse gender needs, address women’s rights and access to justice, strengthen women’s participation in administrative and political processes and address gender inequalities in macro-economic policy formulation, and implementation and evaluation (GOU, 2007).

The NGP has been reflected in development programmes including the third National Development Plan (NDPIII) 2020/21 – 2024/25. The NGP stresses that all national programmes must advance equal gender relations by eliminating gender discriminatory practices, norms and values, preventing and responding to gender-based violence, executing sexual and reproductive health rights programmes, implementing legal literacy programmes for women, supporting social protection for vulnerable women, promoting time-saving technologies, developing incentive frameworks for poor women and eradicating exploitation of women and girls.

## Methods

This article is based on policy documents and published literature on COVID-19 and its impact on women and girls. It draws on official GOU documents about the management of COVID-19, international human rights instruments, national health and development policy documents, WHO publications and reports and journal articles covering COVID-19 and its impacts on women and girls. The majority of these documents were accessed from the government of Uganda websites. The first search for journal papers resulted in thirty-eight (38) papers but only nineteen (19) that were judged to be of high quality were included in the review covering Africa and other parts of the world. Literature published between 2005 and 2022 was considered for inclusion with a high number focused on the period 2020 and 2022 when the COVID-19 pandemic was confirmed.

The policy documents were analysed using content analysis (Bryman, 2001). The process involved a systematic examination of literature to identify concepts and key themes such as the impact of COVID-19 on women’s rights that were relevant to the research topic. The key concepts and themes formed the units of analysis, and these were then used to identify implications for local and international practice. Only documents written in English were reviewed and analysed.

## The Management of COVID-19 in Uganda

Uganda confirmed the first COVID-19 case in March 2020, and the number of cases has since then increased to 163,301 as of March 2022 (Ministry of Health (MOH), 2022). Following WHO guidance, the GOU through the Ministry of Health passed the Public Health Control of COVID-19 Rules (Under Sects. 11 and 27 of the Public Health Act, Cap. 281). There were directives to close schools and institutions of higher learning, business and trading centres and regulation of work in markets (Government of Uganda, 2020). For instance, whereas markets were not completely closed, they were allowed to sell only foodstuffs. Any other trading of non-food items such as the sale of items like clothes, necklaces, shoes and informal items dominated by women was banned.

In addition, all government ministries, agencies and departments were directed to work out plans to ensure that only essential staff would physically report for duty in the offices. Additional public health measures to contain the pandemic were instituted such as the closure of the country’s borders, quarantining of individuals (restriction of movement, or separation from the rest of the population) of healthy persons who may have been exposed to the virus and immediate suspension of public transport to minimise movement and contact among people. (Ministry of Health (MOH), 2020a, b). To reduce infection rates, the general population was advised to minimise social contact by staying and working from home. These control measures uncovered the gendered effects of the COVID-19 pandemic on women’s rights and wellbeing.

## Women’s Rights and Gender Equality

The GOU recognises gender inequality as a constraint to growth in the country (NPA, 2020). Gender equality refers to equal valuing by the public of the similarities and differences of women and men and their roles. This is well-aligned with SDG 5 which is aimed at achieving gender equality and empowering all women and girls in all contexts.

To ensure growth and prosperity, states are required to pursue progress in a fair way that grants women equal rights with men in the political, social, economic and cultural life of their countries. Gross inequalities in work and wages, unpaid women’s work, discrimination in public decision-making, violence and exploitation of women and girls, harmful practices and unequal access to universal reproductive rights and health need to be exposed and eliminated. Elimination of these would foster equal rights and empowerment of women and lead to sustainable and inclusive development.

## The Implications of COVID on Women’s Economic Rights

The National Development Plan III (2020/2021–2024/2025) guides the establishment of strategies to increase household incomes and improve the quality of life of all people. Women have equal rights as men, to participate in the economic life of their countries at all times and in all contexts. Under this initiative, States must create an enabling environment where women and men are empowered to join gainful employment. This obligation requires a gender sensitivity lens to recognise different situations and needs of women and men throughout the decision-making processes. For instance, some women may require affirmative action to balance their roles both in the monetised and non-monetised sectors of the economy.

However, Uganda’s COVID-19 response measures did not take into account the economic and livelihood needs of women and girls. Previously, there has been marked gendered segregation of Ugandan women in the formal sector with many being concentrated in client-facing roles, leisure, travel and hospitality business (Akina Mama wa Africa, 2020). This means that majority is absorbed in the informal economy. The COVID-19 restrictions on movement in Uganda exacerbated the fragility of women’s livelihoods who largely depended on the informal sector. The closure of these small-scale businesses and the prohibition of transportation in Uganda resulted in a major loss of jobs, income and homes (Bukuluki et al., 2020; PGHJG & Bukuluki, 2021). Restriction of movement, closure of markets, loss of jobs and curtailed opportunities to earn a livelihood indicate that the vast majority are at risk of falling back into poverty (Burki, 2020).

The United Nations Development Programme (UNDP) (2020b) revealed that 46% of workers employed in informal businesses (such as hospitality and trade) were pushed below the poverty line during the lockdown. The maintenance of curfew rules left women with limited or no options for survival including limited access to food, health, education, employment and other needs. Women’s sources of income were disrupted because they could no longer access the marketplaces to sell their products and engage in petty trade. In addition, the confinement of potential consumers at home and the closure of borders and markets limited trading opportunities (Women's International Peace Centre et al., 2020).

Although an economic shutdown of predominant female sectors has affected women in both developed and low-income states (Peck, 2020), women in poor countries face additional challenges because, in part, they lack training and income-generating opportunities and alternative livelihood assets such as land. Despite calls by the WHO for governments to weigh the effects of imposed lockdowns on the poor, many countries prioritised containment of the pandemic (Akina Mama wa Africa, 2020; UN Women, 2021).

## COVID-19 and Its Effects on Women’s Unpaid Work

Uganda’s National Equal Opportunities Policy 2006 envisioned a just, free and fair development process and establishment of affirmative action where imbalances exist (Ministry of Gender, Labour & Social Development, 2006). However, the COVID restrictive measures have escalated the unequal gendered distribution of labour and domestication (a significant burden of increased housework and unpaid care) of women in Uganda (Akina Mama wa Africa, 2020). Before the emergence of COVID‐19, women across the world faced context-specific home-based care and work challenges (Peck, 2020). Uganda’s Strategic Country Gender Assessment (SCGA) found that women worked longer hours compared to men (between 12 and 18 h a day, with a mean of 15 h), compared with an average male working day of 8 to 10 h (World Bank Uganda, 2005). Women have a continuous shift workload in both productive work and household tasks (such as caring for children, the elderly and the sick, fetching water and firewood for cooking and meal preparation). Most of these roles are unpaid and reflect harsh choices and trade-offs on women’s time (World Bank Uganda, 2005).

Though imbalanced gender division of labour has been a continuing trend, COVID-19 and its effects have exacerbated women’s workload. For instance, Lutamaguzi and Nanozi (2020) assert that the Ugandan women’s burden of care increased as health and social systems were struggling to cope with rising patient caseloads. Given the biased cultural values and prevailing unequal gender division of labour in favour of men, the women continued to care (looking after the physical, psychological, emotional and developmental needs) of large families that were confined in homes, especially out-of-school children. This increased burden of women’s care work also blocked their performance and representation in paid productive work as they needed to balance paid work and domestic work. This reiterates Esser et al. (2020) research from ten countries across Europe that reported similarities in the discriminatory burden of care-work on women. For instance, they assert that in Spain, challenges of increased tasks for carers (mothers of young children or those with sick dependents in the household) affected their productive work (Esser et al., 2020).

In the USA, more women (14%) compared to men (11%) left their jobs/careers to attend to household needs (Peck, 2020). Though this affects women in most contexts, social services in developed countries have measures to support people with care roles such as cash payments for caretakers (UN, 2020). In a large part of the developing world, this is an expected duty that women must conduct without payment which deprives women of time for full and effective participation in development.

## COVID-19 Implications on Women’s Health Needs

The pandemic has worsened maternal health outcomes globally. Uganda has registered an increase in maternal deaths due to involuntary confinement and resultant financial uncertainty (Akina Mama wa Africa, 2020). Uganda as a low-income country has previously grappled with a fragile health system characterised by systemic infrastructure and human resources challenges (Mwenyango, 2020; Women’s International Peace Centre et al., 2020). The COVID- 19 response measures aggravated this health situation and affected a realisation of the specific health needs of women and girls.

Restrictive stay-at-home measures and a ban on public transport increased cases of reproductive coercion/ sexual abuse and inadequate access to essential reproductive health services. Many women were intimidated into abandoning contraception (the pill, a condom and intra-uterine device) (Lutamaguzi & Nannozi, 2020). The lockdown reduced access to services such as reproductive, maternal, newborn and child health interventions including emergency obstetric care due to the ban on cheap public transport (PGHJG & Bukuluki, 2021).

Likewise, there has also been a global increase in maternal deaths, stillbirth, ruptured ectopic pregnancies and maternal depression due to a neglect of sexual and reproductive health services in some countries such as Mexico, India and the UK (Chmielewska et al., 2021).

PGHJG and Bukuluki (2021) assert that even when the government relaxed restrictions, public transport costs increased to 5 times the normal price making it difficult for health service providers to reach health facilities and for women to access health services. This correlates with research in India that found a huge decline in the number of women registering for antenatal care because public sector personnel was extremely engaged with COVID-19-related work (Vora et al., 2020).

COVID-19 control measures also contributed to sexual abuse and increased teenage pregnancies possibly due to inadequate access to facilities for menstrual hygiene management and lack of basic requirements. An investigation by the Women’s International Peace Centre et al. (2020, p. 7) found a sharp increase in teenage pregnancies (10–19 years) in various Ugandan districts during the lockdown; for instance, 1519 were recorded in Kitgum, 2618 in Kabale and 2598 in Yumbe between January and July 2020.

A situation analysis of the impact of COVID-19 on school-going girls and young women in Uganda by the United Nations Children's Fund (UNICEF) reported a 22.5% increase in pregnancy among girls aged 10–24 seeking first antenatal care from 80,653 to 98,810.^2^ Unmet need for specific health services for women and girls (such as vaccines and reproductive health care) exacerbates maternal mortality and morbidity and increases the risks of early and unwanted pregnancies, HIV and other sexually transmitted diseases.

## COVID-19 and Gender-Based Violence (GBV)

The National Policy on Elimination of Gender-Based Violence in Uganda aimed to eliminate gender-based violations and impunity (Ministry of Gender, Labour & Social Development, 2016). One of the indirect impacts of the pandemic on women and girls has been aggravated gender-based violence (GBV) in the form of threats, coercion and arbitrary deprivations of liberty, especially in the private sphere. Reports from Uganda indicate that physical violence and emotional abuse against women increased (Lutamaguzi & Nannozi, 2020; MOFPED, 2020) partly because men lost their traditional provisional role.

Akina Mama wa Africa (2020) reports that the country recorded an increase in domestic violence caseload against women and children, with at least 3280 cases of gender-based violence in just a few months of announcing the COVID-19 restrictions.

Likewise, in France, cases of domestic violence increased by 30% in the first week of instituting its lockdown, Cyprus and Singapore registered an increase in helpline calls by 30% and 33%, respectively and Argentina registered a 25% increase in domestic violence emergency during the lockdown (United Nations Development Programme (UNDP) (2020a); UN Women, 2020; Burki, 2020).

In the public sphere, market women were forced to sleep on the job due to curfew with some being humiliated, injured and arrested by security personnel (Ainamani et al., 2020).

Although GBV is prevalent in most societies (Gardsbane et al., 2021; Lutamaguzi & Nannozi, 2020), the pandemic and subsequent responses increased it in various forms (domestic violence, sexual harassment, rape and defilement). Globally, 243 million women and girls between 15 and 49 were subjected to sexual and/or physical violence by an intimate partner in 2020 (UNDP, 2020).

GBV against women and girls during the pandemic was amplified by financial difficulties, confined living conditions, movement restrictions and deserted public spaces (UN Women, 2021). GBV has serious consequences for women’s and girls’ mental and physical wellbeing and reduces their productivity (Bukuluki et al., 2020; Mwenyango, 2020). The restrictions on the movement of people and vehicles implied that victims were neither able to access legal, health or psychosocial services as well as safety and security as stated in the National Policy on Elimination of Gender-Based Violence.

## COVID-19 Implications on the Young Women’s Right to Education

Young women’s and girls’ rights to education and literacy were risked during the lockdown. When education institutions in Uganda were closed, over 15 million learners (half of these being girls and young women) suffered (Ministry of Finance, Planning and Economic Development (MOFPED), 2020). However, prolonged stay of girls within communities exposed them to sexual exploiters/perpetrators (close relatives or their peers) and gender-stereotyped home-based care which deprived them of revision time. Gender restrictive social norms also cut them off from their social networks and social support system and access to sexuality education and youth-friendly family planning services (PGHJG & Bukuluki, 2021).

The decision to close schools possibly saved learners worldwide (NPA, 2021); however, the government needed contingency plans to ensure continuity of education not only for the urban children but also for the rural and poor children with limited access to remote learning technologies. The government reopened (on 10th January 2022 after 83 weeks’ closure) and instructed schools to readmit pregnant or young mothers. Given the paucity of psychosocial support in schools and the lack of adequate preparation for both teachers and students, this new normal will be a huge challenge. Furthermore, it is anticipated that 30% of learners will not return to school forever due to teenage pregnancies, early marriages, child labour and stigma (NPA, 2021; Women’s International Peace Centre et al., 2020) despite the “the go back to school and stay in school campaign” strategy alluded in Gender in Education Sector Policy (2016). This is not only against Convention of the Rights of Child (CRC) article 28 which stresses the right to equitable education opportunities but is also likely to jeopardise the country’s future (especially with fewer employment opportunities for untrained women) given the importance of education and literacy as human development indicators.

## COVID-19, Discrimination and the Legal Protection of Women’s Rights

Article 2 of the CEDAW imposes an obligation on states to establish effective legal protection of the rights of women on an equal basis with men. Women need protection from exploitation and discrimination especially because emergencies intensify violence and disrupt existing protective structures (Lutamaguzi & Nannozi, 2020; UN, 2020). Imposing the lockdown limited women’s movement and isolated them from social support systems (such as religious leaders) amid increased financial strains, household tensions, psychological distress and GBV cases.

Women and girls have been highly exposed to discriminatory economic, political, social and cultural practices such as poverty, school dropout, unintended pregnancy or early marriage (to meet household needs) and sexual harassment (MOFPED, 2020; PGHJG & Bukuluki, 2021). For example, the rate of intimate partner physical violence against women increased from 46% before the lockdown to 56% in the first phase of the lockdown (Women’s International Peace Centre et al., 2020). The major challenge is that victims often cover up violence due to limited access to information services and norms and values that instil fear among victims, social stigma and costs of following cases (Gardsbane et al., 2021).

The pandemic risked women’s rights because services in different ministries and institutions including the health sector, justice sector, internal security, local government (districts) and non-government organisations services were disrupted due to limited financial and human resources. This restricted monitoring, evaluation and coordination efforts for apprehending perpetrators and support to victims. For instance, at a certain point, MoGLSD lacked funds to support GBV prevention and response while the closure of the court system (except for capital offences) delayed justice and GBV-related legislation (PGHJG & Bukuluki, 2021). Failure to protect women and girls’ rights leads to normalisation of rights violations and intensifies health complications and economic consequences for individuals and communities.

Discrimination against women was noticeable in the political realm during the pandemic. Despite a universal obligation on states to establish equal opportunities and strategies for full and effective participation of women in leadership, rarely has this goal been met even before the pandemic (Peck, 2020). Like other countries, Uganda held general elections for the presidency, members of parliament, local councils and interest groups during the pandemic (January 2021).

Discrimination against women was manifested in the Electoral Commission’s (EC) revised and stringent roadmap. For example, the EC banned political rallies and processions and made the entire process unaffordable for the poor. Expensive restrictions (such as the need to use print and social media) and curfew rules curtailed general participation in these elections (Babirye, 2020). Women, in particular, faced constraints in engaging in elective processes due to pre-existing inequalities, inadequate access to appropriate technology and limited resources for financing scientific campaigns.

## Local and International Implications for Practice

The foregoing discussion shows the gendered effects of the COVID-19 pandemic on women’s economic and education rights, unpaid work and health needs and indicates a resurgence of gender-based violence (GBV) and discriminatory practices against women and girls. This is concerning because they are happening with minimal or no protection because available resources are highly prioritised in the management of the pandemic. This raises an important question: how to stitch these gaps to protect rights and improve the wellbeing of women, girls and other marginalised populations during crises like the COVID-19 pandemic?

At the outset, the GOU, civil society organisations and the international community need to re-examine COVID-19 measures and guidelines and ensure that they are gender-responsive and conform to human rights standards. To ensure that COVID-19 responses are gender-responsive, various social protection measures must be in place to shelter the most disadvantaged (such as the provision of food relief and cash transfers) and most importantly, women must be represented in professional and technical COVID-19 task forces. Given that most of the COVID-19 containment efforts are largely funded by development partners, they should avail sustainable and prompt funding to facilitate the formation of country-specific functional gender-sensitive management committees to promote gender equality initiatives and women’s rights.

It is also important to anticipate the gendered impacts of the lockdowns and stay-at-home orders on women and girls and other vulnerable groups. This is necessary to establish policies, systems, structures and practices that meet their specific needs and priorities, for example, measures to minimise delays in accessing specialised health care (including antenatal and postnatal care, counselling and psychosocial support for GBV survivors) and affordable legal protection. Sustainable recovery strategies may involve recognition of women’s unpaid work, provision of equal opportunities and social protection schemes (UN, 2020).

Furthermore, affirmative action as enshrined in legal and human rights instruments must be pursued to bridge gender gaps and address historical and present forms of discrimination against women and girls in the economic, social, political and cultural spheres. For instance, social security protection measures to cover informal workers or capital and economic stimulus packages and emergency measures as implemented in other countries (UN, 2020) can be adapted to the Ugandan context to mitigate the impacts of COVID-19 on women in the informal sector who have been adversely affected by lockdowns and restrictive COVID-19 measures. Sensitisation campaigns are necessary to address discriminatory social and cultural practices (such as early marriage). For example, seminars and workshops through media platforms can help ensure the safety of women and girls in communities.

The role of professionals in different ministries and institutions including the health, education, justice, internal security sectors and local governments must not be overlooked. This research has revealed several issues including violation of women’s rights, gender-based violence and discrimination and their consequences on individual women and national development. Health workers, social workers, counsellors, lawyers and other professionals must provide seamless practice in terms of needs assessments, information sharing, management and referral. For example, probation officers are versed in human rights. They could form useful partners for assessing risks associated with response measures (such as lockdown and restrictions on movement) and monitoring the COVID-19 situation in communities and providing required individual and group counselling.

Professionals over the world need to innovate ways of advocacy to ensure that the voices of disadvantaged groups reach COVID-19 management task forces in all contexts, for example, social workers can aid the transformation of the education system to integrate teaching methods that can address the special needs of traumatised or teenage mothers. This will ensure prioritisation of their rights and needs for safety, health and wellbeing and foster inclusive development in the long run.

## Conclusion

Gender equality is not only a right but also a necessity for the achievement of further development goals. The COVID-19 containment measures adopted by GOU have impaired women’s rights and escalated unequal power relations causing high levels of violence against women and girls, death, disability, teenage/early pregnancies and school dropouts. Mandatory lockdowns and movement restrictions created negative implications for women’s attainment of economic, social, cultural, political and civil rights and intensified pre-existing gender inequalities between women and men.

The oversights (in the design and implementation of COVID-19 control measures) discussed in this article are great lessons for upgrading the management of ongoing and future pandemics. Uganda’s government planners and development partners need to design and implement comprehensive and integrated response services to safeguard the wellbeing and survival of vulnerable populations. Such interventions must also consider and address their routine challenges.

This research has indicated a need for policymakers, community leaders and service providers to apply gender analysis in the delineation of public health measures and standard operating procedures on women’s wellbeing and rights.

## Endnotes

https://www.health.go.ug/

https://www.unicef.org/uganda/press-releases/prioritize-re-opening-schools-secure-childrens-well-being
